# The relationship between iodine intake and the risk of thyroid cancer: A meta-analysis

**DOI:** 10.1097/MD.0000000000006734

**Published:** 2017-05-19

**Authors:** Ling-Zhi Cao, Xiao-Dong Peng, Jian-Ping Xie, Fan-Hui Yang, Hu-Ling Wen, Suping Li

**Affiliations:** a Department of Nuclear Medicine, Affiliated Hospital of North Sichuan Medical College, Nanchong; b Key Laboratory of Chronobiology of the Health Ministry, Basic and Forensic School, Sichuan University, Chengdu, Sichuan, China.

**Keywords:** iodine, meta-analysis, risk factor, thyroid neoplasms

## Abstract

Thyroid cancer (TC) is the most common malignancy of the endocrine system. The relationship between iodine intake and TC risk is controversial always. We aim to figure out the relationship between iodine intake and TC using meta-analysis. Literature research in MEDLINE, Embase, China National Knowledge Infrastructure, and China BioMedicine was performed up to April 2016, searched for relevant case–control and cohort studies. The effect of iodine consumption on the risk of TC was assessed using the pooled odds ratio (OR) and 95% confidence interval (CI). The meta-analysis included 8 case–control studies (n = 4974; 2213 cases; 2761 controls). More than adequate or excess iodine intake (>300 μg/d) decreased the risk of TC (OR 0.74, 95% CI 0.60, 0.92). High consumption of saltwater fish or shellfish decreased the risk of TC (OR 0.72, 95% CI 0.55, 0.95; OR 0.70, 95% CI 0.52, 0.96; respectively). A higher intake of dietary iodine was as a protective factor for TC. However, the available data are very limited and more studies are required.

## 1 Introduction

Thyroid cancer (TC) is the most common malignancy of the endocrine system.^[1]^ The incidence of TC is increasing in many countries.^[2]^ Data published in 2014 showed that TC had become the fifth most common cancer among women in the United States.^[3]^ In North America, the incidence of TC has been increasing by more than 6% a year, and the proportion of papillary carcinoma has increased significantly, from 58% (1970–1980) to 85.9% (2000–2010).^[4]^

Iodine is an essential component for thyroid hormone synthesis. There is a U-shaped relationship between iodine intake and thyroid disease, indicating that either too much or too little iodine intake will lead to thyroid diseases.^[5]^ Goiter has been associated, for a long time, with either high iodine intake or chronic iodine deficiency, and goiter has been shown to be related to an increased risk of TC.^[6]^ Various studies have shown different results for the relationship between iodine and TC. For example, a higher dietary iodine intake has, in fact, been shown to significantly decrease the risk of TC.^[7]^ Conversely, a case–control study from Hawaii indicated that a high consumption of seafood (food rich in iodine) increased the risk of TC in women.^[8]^ Meanwhile, another study indicated that there was little association between consumption of seafood and TC risk.^[9]^

It remains considerable controversy about the relationship between iodine intake and TC risk. Therefore, we performed current meta-analysis of all available epidemiological studies to provide a comprehensive estimate of the possible relationship between iodine intake and TC risk.

## 2 Materials and methods

### 2.1 Ethics statement

This study is a meta-analysis and ethics statement is not applicable.

### 2.2 Literature search

MEDLINE, Embase, China National Knowledge Infrastructure (CNKI), and China BioMedicine (CMB) were searched by computer from the inception of each database to April 2016. In addition, the reference lists of relevant articles were searched to identify additional relevant papers. The searches of the MEDLINE and Embase databases used the following search terms: (“thyroid neoplasm” OR “thyroid cancer” OR “thyroid carcinoma” OR “thyroid tumor”) AND (“iodine not iodine radioisotopes” OR “seafood” OR “sea food” OR “iodine intake” OR “dietary iodine”) AND (“case control” OR “case-control” OR “cohort study” OR “clinical trial” OR “observational study”). The search terms “thyroid cancer and risk factor” (in Chinese) were used to search the Chinese CNKI and CMB databases, and then the results were further searched using “iodine or seafood” (in Chinese).

### 2.3 Inclusion and exclusion criteria

The inclusion criteria included: pathological type: TC; study design: case–control studies or cohort studies; iodine exposure: the specific amount of iodine intake and iodine-rich foods. Exclusion criteria were study without adequate data, animal studies, case report and reviews.

### 2.4 Data extraction and quality evaluation

Data were extracted from the included studies by 2 authors (L-ZC and H-LW) independently. If necessary, consensus was achieved by discussion and reexamination. The following data were extracted from eligible studies: surname of first author, study design, location, study population, iodine exposure. The Newcastle–Ottawa Assessment Scale (NOS) was used to score the included studies for methodological quality,^[10,11]^ with the highest quality studies being awarded a maximum score of 9. This assessment was independently performed by 2 authors (L-ZC and H-LW), with a third author (X-DP) being consulted to settle disagreements.

### 2.5 Statistical analyses

The odds ratio (OR) and 95% confidence interval (CI) were the statistical effect size used to estimate the effect of exposures. The *I*^2^ test was used to quantify heterogeneity.^[12]^ According to the Cochrane Handbook for Systematic Reviews,^[13]^ as long as *I*^2^ was less than 50%, heterogeneity could be accepted, and the fixed-effects model was used. When high levels of heterogeneity (*I*^2^ > 50%) were detected between the studies, the random-effects model was selected. When different subgroups in the same group required different models, the random-effects model was used, which was as suitable as the fixed-effects model as it would not substantially change the results. Publication bias was assessed using a funnel plot for asymmetry for the group of more than 10 studies.^[14]^ The pooled OR and 95% CI were recalculated using the “Tim and Fill” procedure after imputation of the results of hypothetically missing studies that would be needed to minimize the effect of publication bias.^[14]^ The OR and 95% CI were estimated by Review Manager software, version 5.2 (The Cochrane Collaboration, Oxford, UK). A *P* value <.05 was considered statistically significant.

### 2.6 Iodine exposure measures

Data on the specific amount of iodine intake were classified according to published standards: <30 μg/d (severe deficiency); 30 to 74 μg/d (moderate deficiency); 75 to 149 μg/d (mild deficiency); 150 to 299 μg/d (optimal reference); 300 to 449 μg/d (more than adequate); and >450 μg/d (possible excess).^[15]^ The consumption of seafood was classified as either low (<1 time/wk or <4 times/mo), moderate (≥1, <3 times/wk or ≥4, <12 times/mo), or high (≥3 times/wk or ≥12 times/mo).

## 3 Results

All of the included studies were case–control studies, which retrospectively assessed iodine exposure using questionnaires. Of a total of 3566 papers identified from the initial searches, 23 studies were identified to be potentially eligible for inclusion after reviewing titles and abstracts. Of these 23 full-text papers, 13 studies were deemed to be suitable. Five studies were excluded from the 13 full-text papers because they were pooled analyses. Thus, there were 8 studies included in the meta-analysis (n = 4974; 2213 cases; 2761 controls) (Table 1).^[6,7,9,16–20]^ The NOS scores of the included studies ranged from 4 to 7, with a median score of 6 and a mean score of 5.5. Studies with a score of at least 6 were considered as being of high quality. Five (62.5%) studies were deemed to be of high quality (Table 1). Lower quality scores primarily arose from ascertainment of exposure and nonresponse rate, with the definition of control also affecting the quality.

**Table 1:**
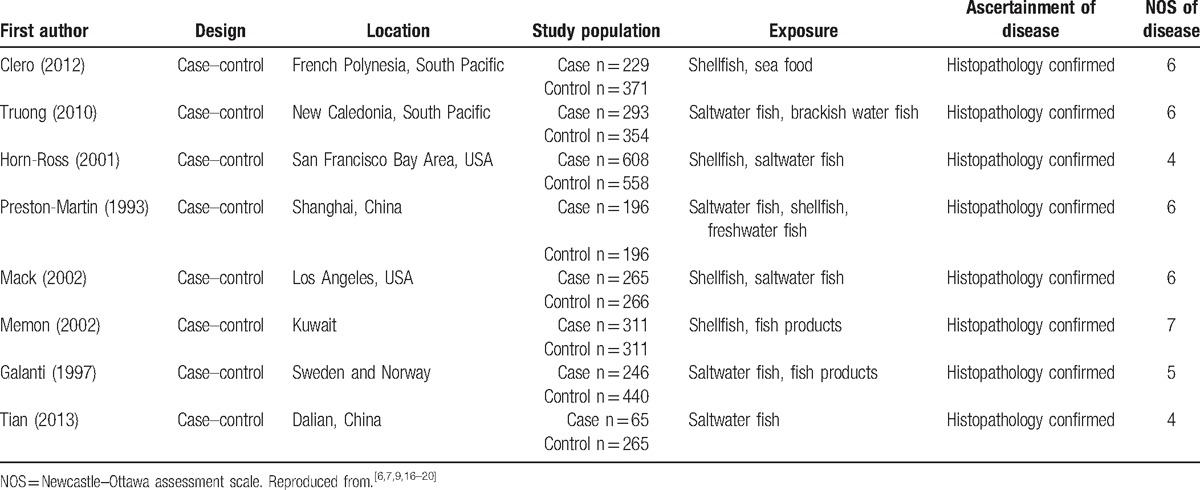
Characteristics of studies included in a meta-analysis undertaken to estimate the possible relationship between iodine intake and TC risk.

**Figure 1:**
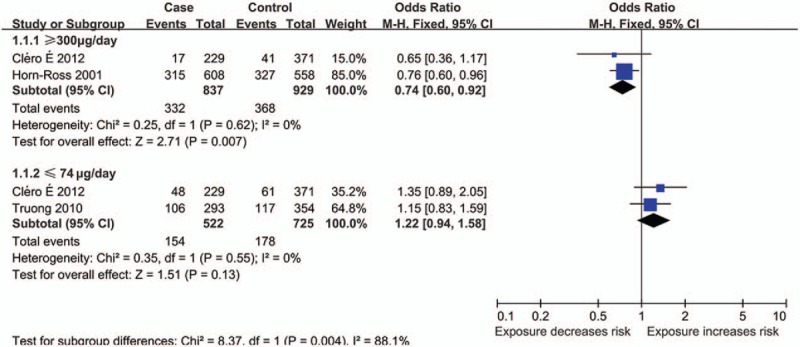
Forest plot for the effect of iodine intake (≥300 and ≤74 μg/d) on the risk of TC. CI = confidence interval, M–H = Mantel–Haenszel. Reproduced from.^[6,7,9]^

Three studies calculated the specific amount (μg/d) of iodine intake.^[6,7,9]^ Their indexing standards were not the same, but parts were the same. In current meta-analysis, the same parts of the data were pooled analysis. The results showed that more than adequate and possible excess of iodine (≥300 μg/d) could decrease TC risk (OR 0.74; 95% CI 0.60, 0.92; *P* = .007)^[6,7]^ (Fig. 1) but showed the relationship between moderate and severe deficiency of iodine (≤74 μg/d), and TC risk was not statistically significant (Fig. 1).^[7,9]^

**Figure 2:**
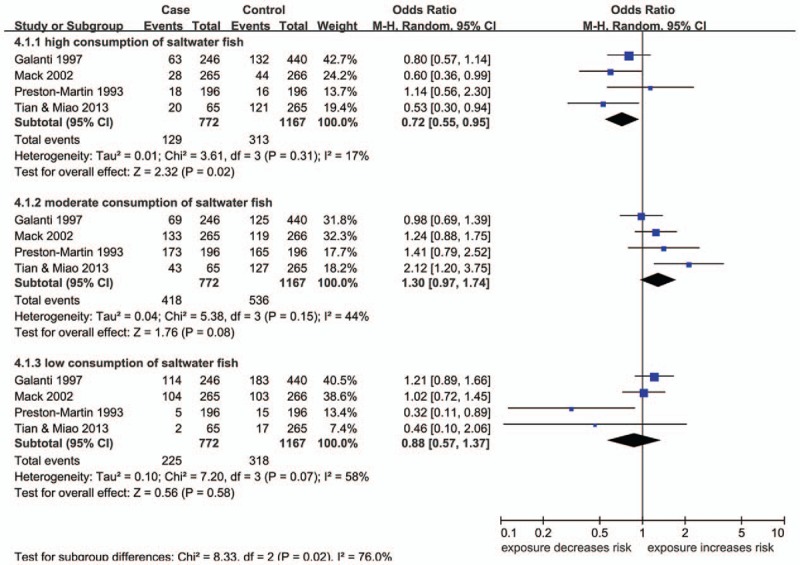
Forest plots for the effect of different levels of consumption of saltwater fish on the risk of TC. CI = confidence interval, M–H = Mantel–Haenszel. Reproduced from.^[16–19]^

A total of 4 articles are studied on the relationship between saltwater fish and TC risk.^[16–19]^ The results showed that high consumption of saltwater fish (≥3 times/wk or ≥12 times/mo) could decrease TC risk (OR 0.72; 95% CI 0.55, 0.95; *P* = .02) (Fig. 2). However, the analysis showed that the relationship between moderate or low consumption of saltwater fish and TC risk was not statistically significant (Fig. 2).

**Figure 3:**
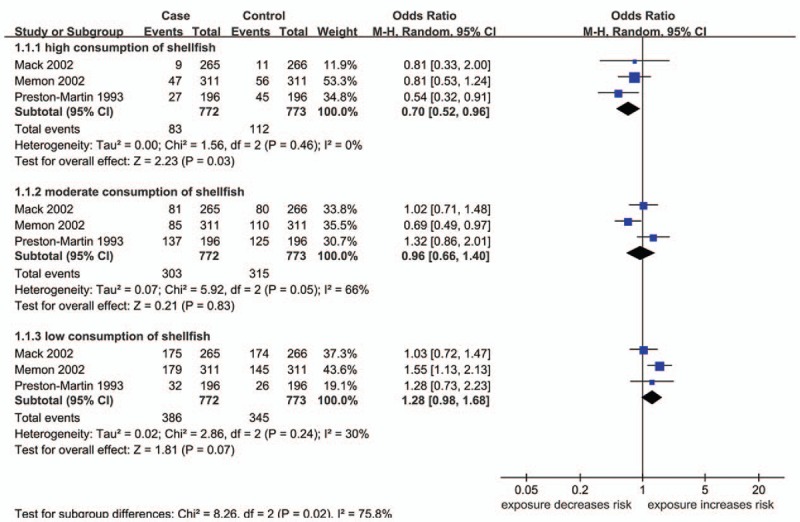
Forest plots for the effect of different levels of consumption of shellfish on the risk of TC. CI = confidence interval, M–H = Mantel–Haenszel. Reproduced from.^[16,17,20]^

Three articles are studied on the relationship between shellfish and TC risk.^[16,17,20]^ Subgroup analysis indicated that high consumption of shellfish (≥3 times/wk or ≥12 times/mo) could also decrease the risk of TC (OR 0.70; 95% CI 0.52, 0.96; *P* = .03) (Fig. 3). However, the relationship between moderate or low consumption of shellfish and TC risk was not statistically significant (Fig. 3).

With regard to publication bias, owing to the limited number (below 10) of studies included in the saltwater fish, shellfish, and iodine intake analyses, publication bias was not assessed.

## 4 Discussion

The TC incidence has been rising in recent years, although the increase incidence is at least in part due to more sensitive screening. This is the first meta-analysis to focus on the relationship between a specific amount of iodine intake and TC risk. This present meta-analysis demonstrated that a higher iodine intake (≥300 μg/d) and high consumption of saltwater fish and shellfish were protective factors for TC. All of the patients in the included studies were identified by histopathological diagnosis. The data included in this present study were included after a comprehensive search of the published literature, but the analysis remained limited because there were only 3 studies that calculated the specific amount of iodine intake.^[6,7,9]^ All of these studies used CIOUAL a validated French food composition table for calculating the iodine content in food.^[6,7,9]^ The classification standards of iodine nutrition were different in the 3 studies, but parts were the same, which allowed for the pooled analysis of the common parts.^[6,7,9]^ The other reports included in this present meta-analysis did not calculate the specific amount of iodine content in the diet but instead considered the consumption frequency of seafood (saltwater fish and shellfish). Saltwater fish and shellfish are the common iodine rich food and the main source of dietary iodine. The iodine content of shellfish is the greatest with more than 120 μg/100 g.^[7]^ The results of the analysis of a high consumption frequency of saltwater fish and shellfish were consistent with the results of the higher iodine intake analysis, which underlined the potential function of high iodine intake in protecting against TC. A high consumption frequency of saltwater fish and shellfish can indirectly reflect the level of dietary iodine intake. Further investigations that calculate the specific amount of dietary iodine content in the same way and with the same classification standard of iodine nutrition are needed to increase the reliability of the results.

This present study had several limitations. The searches were restricted by language as they were only conducted using English and Chinese language literature databases, and there was only 1 eligible Chinese article. Moreover, a lack of other language databases did not allow for the inclusion of all relevant articles. All included studies were retrospective case–control studies, which could not avoid recall bias. Cohort studies provide the best evidence for etiological research but no cohort studies were identified in this present meta-analysis and used just saltwater fish and shell fish as indicator of iodide intake.

In conclusion, these current meta-analysis results suggest that a higher dietary iodine intake or high consumption of shellfish and saltwater fish have a protective effect against TC in populations mainly based in coastal cities or on islands. This report can serve as the basis of more in-depth studies in the future.
